# Society Meetings

**Published:** 1901-02

**Authors:** 


### SOCIETY MEETINGS.

THE Medical Union held its nineteenth annual meeting and banquet at the Genesee Hotel, Wednesday evening, January 23, 1901. The following-named members were present: Drs. A. H. Briggs, R. L. Banta, Vertner Kenerson, S. S. Green, A. E. Diehl, W. S. Renner, S. G. Dorr, J. C. Thompson, De Witt C. Green, H. D. Ingraham, Chas. E. Congdon, L. B. Dorr, W. D. Greene, Brown, Fell, J. H. Potter, Rasbach, Geo. H. Westinghouse and Frederick Preiss. Letters of regret were read from Drs. Wende, Phelps and Park. The following are its present officers: President, Dr. J. H. Potter; vice-president, Dr. F. B. Rasbach; secretary and treasurer, Dr. Frederick Preiss; executive committee, Maj. A. H. Briggs, Dr. R. L. Banta, and Dr. Frederick Preiss.

---

THE Buffalo Academy of Medicine held meetings during the month of January, 1901, as follows:

*Section on Medicine.*—Tuesday evening, January 8th. Program: Arterio-sclerosis, DeLancey Rochester; Reminiscences of malaria, D. A. Morrison.

*Section on Pathology.*—Tuesday evening, January 15th. Program: Sarcoma and sarkoid growth of the skin, J. C. Johnson, assistant pathologist, Cornell University; Urticaria, Grover Wende.

*Section on Ophthalmology, Otology, Rhinology and Laryngology.*—Monday evening, January 21st. Program: The pose of the body as related to the type of the cranium and the direction of the normal visual plane—an anthropological study from an ophthalmological standpoint, George T. Stevens, of New York.

*Section on Obstetrics.*—Thursday evening, January 22d. Program: Symphysiotomy, P. W. Van Peyma; The kidneys and their relation to operations, Stephen Y. Howell.

---

THE Rochester Academy of Medicine—Section on surgery—held its regular monthly meeting in the hall of the Reynolds Library, Wednesday, January 9, 1901, at 8.15 p.m. The following program was carried out: I. Presentation of cases—(*a*) Case of double Colles's fracture, one wrist treated with splints, the other with Moore's dressing, Richard M. Moore; (*b*) Case of comminuted fracture of the patella, William L. Conklin; (*c*) Case of mediastinal abscess, John O. Roe. II. Reports of cases: (*a*) Cancer of cecum, with presentation of specimen, William L. Conklin; (*b*) Case of fracture of the tibia only, Richard M. Moore. III. Paper, A diagnostic sign of complete reduction of fracture at the lower extremity of the humerus, and a method of treatment, Richard M. Moore; IV. Paper, The histological method for the diagnosis of rabies, Charles Wright Dodge. John O. Roe, chairman; E. Wood Ruggles, secretary.

---

The Niagara Falls Academy of Medicine held its regular monthly meeting at the office of Dr. F. Guillemont, 550 Main Street, on Monday evening, January 14, 1901, at 8.45 p.m. A paper was read by Dr. J. H. Miller: subject, Fever, cause and treatment.

